# Noninvasive technique for measurement of heartbeat regularity in zebrafish (Danio rerio) embryos

**DOI:** 10.1186/1472-6750-9-11

**Published:** 2009-02-19

**Authors:** Po Kwok Chan, Chun Chi Lin, Shuk Han Cheng

**Affiliations:** 1 Department of Biology and Chemistry, City University of Hong Kong, 83 Tat Chee Avenue, HKSAR, PR China

## Abstract

**Background:**

Zebrafish (*Danio rerio*), due to its optical accessibility and similarity to human, has emerged as model organism for cardiac research. Although various methods have been developed to assess cardiac functions in zebrafish embryos, there lacks a method to assess heartbeat regularity in blood vessels. Heartbeat regularity is an important parameter for cardiac function and is associated with cardiotoxicity in human being. Using stereomicroscope and digital video camera, we have developed a simple, noninvasive method to measure the heart rate and heartbeat regularity in peripheral blood vessels. Anesthetized embryos were mounted laterally in agarose on a slide and the caudal blood circulation of zebrafish embryo was video-recorded under stereomicroscope and the data was analyzed by custom-made software. The heart rate was determined by digital motion analysis and power spectral analysis through extraction of frequency characteristics of the cardiac rhythm. The heartbeat regularity, defined as the rhythmicity index, was determined by short-time Fourier Transform analysis.

**Results:**

The heart rate measured by this noninvasive method in zebrafish embryos at 52 hour post-fertilization was similar to that determined by direct visual counting of ventricle beating (*p *> 0.05). In addition, the method was validated by a known cardiotoxic drug, terfenadine, which affects heartbeat regularity in humans and induces bradycardia and atrioventricular blockage in zebrafish. A significant decrease in heart rate was found by our method in treated embryos (*p *< 0.01). Moreover, there was a significant increase of the rhythmicity index (p < 0.01), which was supported by an increase in beat-to-beat interval variability (*p *< 0.01) of treated embryos as shown by Poincare plot.

**Conclusion:**

The data support and validate this rapid, simple, noninvasive method, which includes video image analysis and frequency analysis. This method is capable of measuring the heart rate and heartbeat regularity simultaneously via the analysis of caudal blood flow in zebrafish embryos. With the advantages of rapid sample preparation procedures, automatic image analysis and data analysis, this method can potentially be applied to cardiotoxicity screening assay.

## Background

In spite of the morphological differences of the heart, the early function is similar among different model animals studied, including zebrafish [1-3]. The zebrafish embryo has been suggested as an ideal model to uncover the molecular mechanism of cardiac development and to identify genes related to congenital cardiac defects in human [1,4,5]. In addition, although there are differences between zebrafish embryos and human in responses to human cardiotoxic drugs, such as the dissociation between the atrium and ventricle [6,7] in zebrafish embryos which is not normally observed in adult humans, recent studies demonstrated that zebrafish still has some similar physiological responses to those drugs. Bradycardia, one type of cardiac arrhythmia, was induced in zebrafish embryos after treated by well-known QT-prolonging drugs, such as terfenadine [6,8]. Furthermore, a zebrafish ortholog, which encoded for potassium ion channel responsible for ventricular repolarization, was cloned and showed functional similarity with the human counterpart [6].

There are several methods to assess cardiac functions in zebrafish embryos, including the simplest stopwatch counting [9], micro pressure system [10], Laser Doppler microscope technique [11] and electrocardiogram [12]. However, all these techniques are labor-intensive, time-consuming and requires skillful operator to perform the experiments, and thus limits their applications for large-scale studies [11], such as drug toxicity evaluation. Although the new laser-scanning velocimetry [13] can quantify cardiovascular performance in zebrafish embryos, a confocal microscope is needed, which is an expensive equipment and requires skillful operator. Moreover, recent development in digital imaging analysis tools makes analysis of cardiac functions, such as cardiac output [14-18], traveling speed of the blood cells [19,20], visualization and analysis of blood cell distribution, blood cell count and vasculature structure [14,17,21] in transparent zebrafish embryos easier. Recently, Schwerte and his co-workers had developed a non-invasive method to assess heart rate variability in zebrafish embryos [22]. In their method, heart rate was determined from videos of beating heart. In brief, heart signal was filtered by bandpass filter and peak was detected by algorithm fitting quadratic polynomial to signal data. Peak-peak distance was determined and converted to beat-to-beat frequency for plotting cardiotachogram. Variation of heart rate was shown as a prominent scatter around the median values of the cardiotachogram. However, none of the current tools is capable of measuring heartbeat rhythm, an important parameter for cardiac function, from power spectrum of heart signal. Cardiac arrhythmia, i.e. disturbance of heartbeat regularity [23], is associated with cardiotoxicity effect of drugs and sudden cardiac death [24].

This prompts for a methodology applicable in zebrafish embryos to evaluate heartbeat regularity. In the present study, we described a simple, non-invasive methodology by video-recording under stereomicroscope and analyzing the video records with our cuctom made software, which integrated digital motion analysis [20] and power spectral analysis, to determine the heart rate. Short-time Fourier Transform (STFT) analysis was then applied to measure the heartbeat regularity. Furthermore, we verified that treatment by terfenadine, a cardiac toxic drug which induces arrhythmia in human, exhibited atrioventricular blockage in zebrafish embryos as reported previously [6] and showed irregular heartbeat, with increasing variability of heartbeat time interval. The heartbeat regularity in terfenadine-treated embryos determined from power spectral analysis was decreased as compared to control embryos. The results demonstrated that our method is able to assess the cardiac physiology, in term of heart rate and rhythmicity, in zebrafish embryos.

## Results

### Power spectral analysis of caudal circulation in wild type embryos

**Figure 1:**
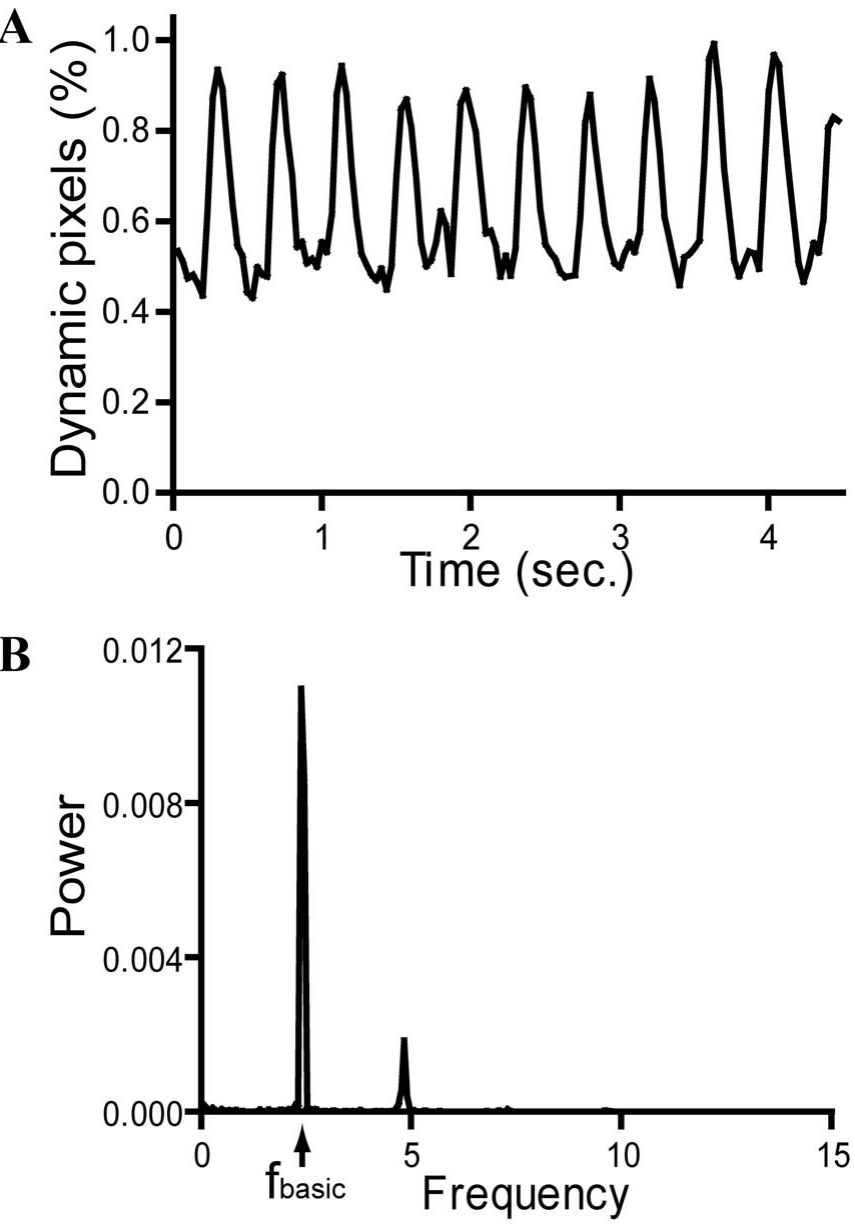
**A typical example of the signal of dynamic pixels (A) and its corresponding power spectrum (B) obtained from the caudal circulation of a 52-hpf control embryo**. f_basic_: first frequency component

Flow of blood cells is clearly seen inside blood vessels in zebrafish embryos once the circulation begins at 24 hpf. The flow is pulsatile, with a rhythm of fast and slow movement. With our image analysis software, this oscillatory movement of blood cells can be visualized by waveform of dynamic pixels. A typical example of waveform of dynamic pixels for a 52 hpf zebrafish embryo is shown in Figure 1A. The waveform exhibited a rapid upstroke of the amount of dynamic pixels followed by a drop, showing the rhythmic change of blood cell velocity. This rhythmic fluctuation of dynamic pixels was consistent with the direct observation of pulsatile flow of blood cells observed under stereo-microscope. Frequency characterization of this rhythmic flow of blood cells was done by transforming the waveform in time domain to frequency domain. Figure 1B showed the corresponding power spectrum for the waveform of dynamic pixels. Two peaks of frequency components (at 2.31 Hz and 4.69 Hz) were identified. The first peak (2.31 Hz) was the basic frequency (f_basic_) component of the waveform of dynamic pixels. The second frequency component was the multiple of the first one and should be considered as the harmonic counterpart of the basic frequency component [25].

### Validation of heartbeat frequency detected by the program

**Figure 2:**
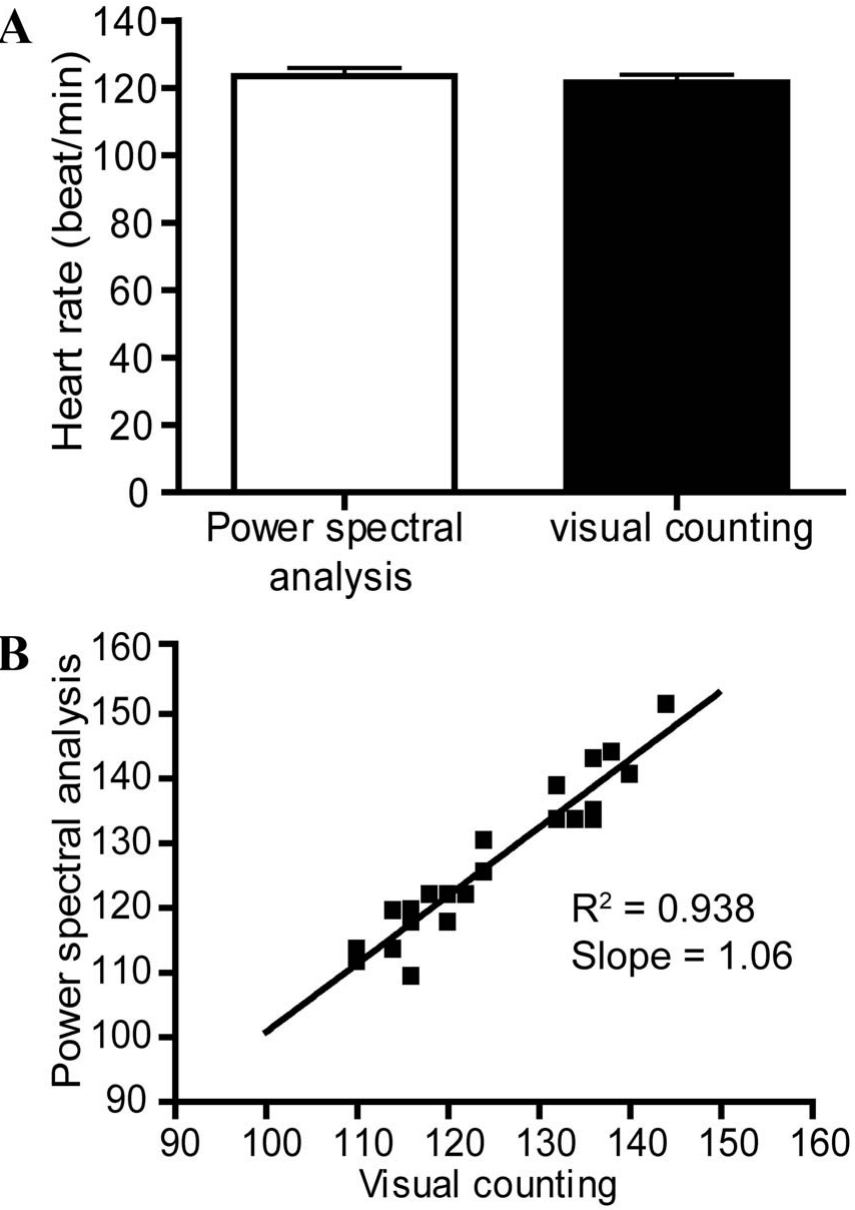
**(A) Heart rate determined from visual examination of heart beats from 52 hpf zebrafish embryos is corresponding to the one determined from power spectral analysis**. (B) Linear correlation between the heart beats analyzed by power spectrum and visual counting was found.

Cardiac performance in wild type zebrafish embryos (n = 25) at 52 hpf were assessed by both power spectra analysis and visual examination (Fig. 2A). The mean value of f_basic _was 2.08 and the heart rate was 125.3 ± 11.9 beat per minute for power spectral analysis. On the other hand, the mean heart rate determined by direct visual examination of ventricle beating was 121.6 ± 11.8 beats per minute. There was no significant difference between them (*p *> 0.05). There was a linear correlation between the heart rate determined by visual inspection and by power spectral analysis and the slope of linear regression was approximately equal to 1 (Fig. 2B). The results indicated that the heart rate determined by our method was equivalent to the heart rate determined by visual counting.

### Demonstration with cardiotoxic drug: terfenadine

Terfenadine, a cardiotoxic drug prominently linked to proarrhythmia in humans, was used as an example. Recent studies showed that terfenadine induced bradycardia and atrioventricular blockage in zebrafish [6,8].

**Figure 3:**
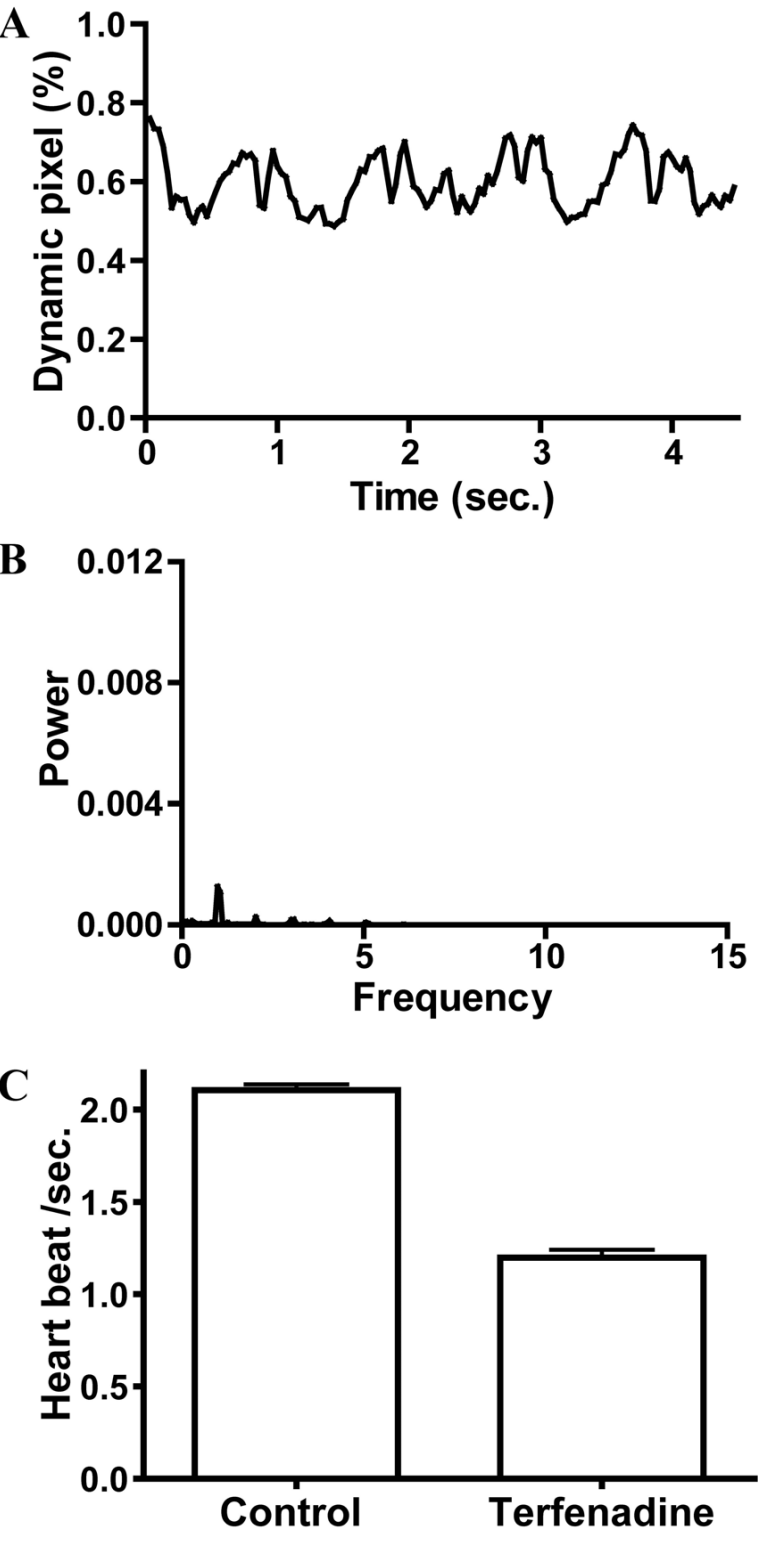
**A typical example of the signal of dynamic pixels (A) and its corresponding power spectrum (B) obtained from the caudal circulation of terfenadine-treated embryo at 52 hpf**. (C) The heart beat frequency of control and terfenadine-treated embryos with bradycardia analyzed by power spectrum. A significant decrease in heart beat frequency was found in terfenadine-treated embryos (*p *< 0.01).

Videos of caudal circulation in terfenadine-treated embryos were analyzed with our program. Direct observation of caudal circulation in terfenadine-treated embryos showed that movement of blood cells in dorsal aorta was pulsatile as seen in control embryos. An example of waveform of dynamic pixels (Fig. 3A) of a terfenadine-treated embryo with severe effect and its corresponding power spectrum (Fig. 3B) were shown. Although the waveform of dynamic pixels also exhibited oscillatory change of blood cells velocity along the time being analyzed, the f_basic _was hard to identify in the power spectrum (Fig. 3B). In general, the f_basic _of terfenadine-treated embryos were just a peak a little bit higher than other frequency components and was less dominant in the power spectra, as compared with the power spectra of control embryos (Fig. 1B).

Terfenadine-treated embryos showed variation of heart beat, from about 1 Hz to 2.5 Hz. (data not shown). Heartbeat frequency of complete terfenadine-treated embryos determined from power spectra was 1.551 ± 0.3633 Hz, which was significantly slower than control embryos (2.165 ± 0.0307 Hz; p < 0.01). The computed heart rate was 93.1 ± 21.8 for terfenadine treatment. We separated the terfenadine-treated embryos into two groups. One group included those embryos with heart beat close to 1 Hz., i.e. bradycardia, while the other group showed normal heart beat as in control. The heart beat frequency (Fig. 3C) of terfenadine-treated embryos with bradycardia (n = 50) determined from power spectra was 1.198 ± 0.0423 Hz, which was significantly slower than that of control embryos (n = 60; 2.165 ± 0.0307 Hz; *p *< 0.01). The computed heart rate was 129.9 ± 11.9 beats per minute for control and 71.9 ± 21.8 beats per minute for terfenadine treatment.

### Increased variation of heart beat interval by terfenadine

**Figure 4:**
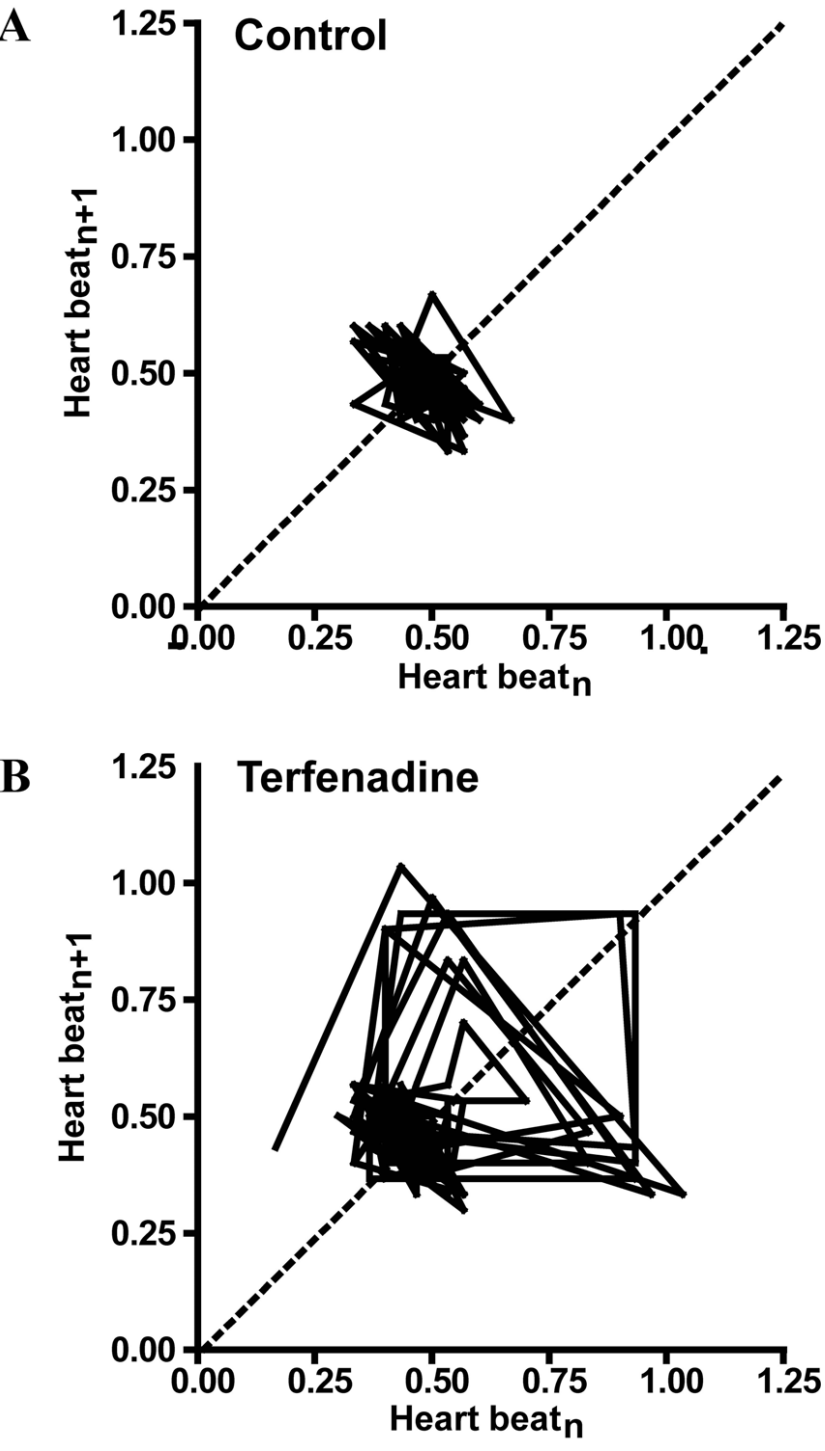
**Poincare plot of time interval of heart beat from (A) control and (B) terfenadine-treated embryos at 52 hpf**. X-axis is the time interval of heart beat at a particular time while Y-axis is the time interval of next heart beat. The shape and size of cluster illustrates the increase of heart beat variability in terfenadine-treated embryos.

Embryos treated with terfenadine showed a significant increase in the variation of heart beat interval. Poincare plot, an emerging quantitative visualization tool, provides additional information about the variation between heartbeats. Typical examples for both control (Fig. 4A) and terfenadine-treated embryos with bradycardia (Fig. 4B) were shown. In control embryo, the plot had concentrated data points arranged from 0.3 to 0.6 sec, clustering around 0.48 sec. In contract, the plot of terfenadine-treated embryos was dispersed, spreading from 0.3 to 1 sec. Although there was a cluster around 0.48 seconds, some of data points were diverged from the cluster, making the standard deviation large. Variation of heart beat interval was significantly increased from 0.0615 ± 0.0107 in control embryos to 0.1857 ± 0.0627 in terfenadine treated embryos (*p *< 0.01), illustrating the increased variation of heartbeat by terfenadine in zebrafish embryos.

### Short-time Fourier Transform (STFT) analysis

**Figure 5:**
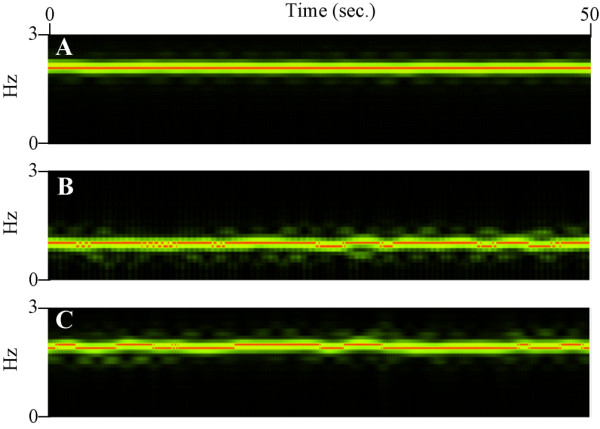
**Short-time Fourier Transform (STFT) analysis**. The figure illustrates the STFT analysis of signal of dynamic pixels in every 0.5 sec along time. The time interval for each analysis is 1 sec. Intensity of green colour is corresponding to the power value while the red colour indicates the f_basic_. (A) Control embryo showed consistent f_basic _during the examination period. In contrast, the f_basic _varied time by time in terfenadine-treated embryos, no matter in embryo with (B) decreased heart rate or (C) normal heart rate.

**Figure 6:**
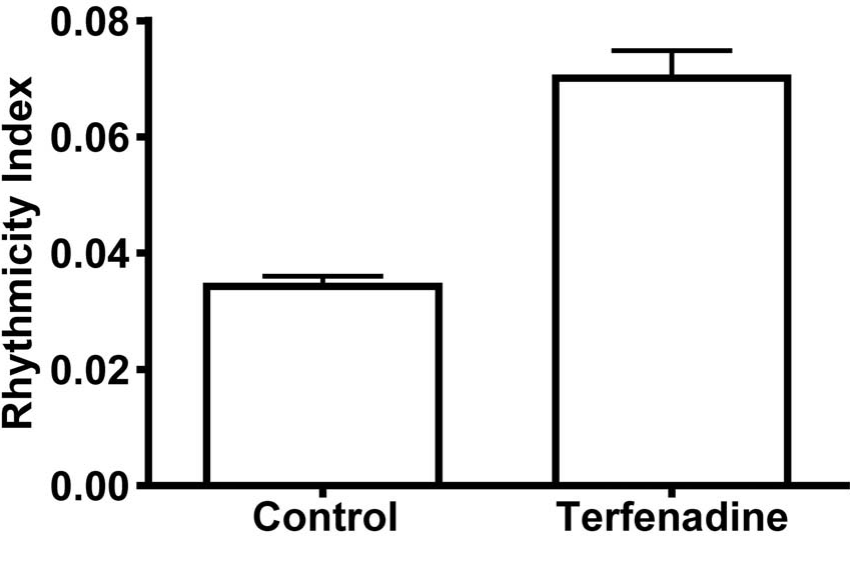
**Effect of terfenadine on heart rate and heart rate variability**. Bradycardia was induced by terfenadine treatment (A). Rhythmicity index was increased by terfenadine treatment (B), suggesting induction of irregularity of heart rate by terfenadine.

Apart from the poincare plot, the variation of the heartbeat was also demonstrated by the changes of f_basic _time by time by STFT analysis by our program. Figure 5 illustrated the changes of frequency by analyzing the signal of dynamic pixels time by time from control and terfenadine-treated embryos. In control embryo (Fig. 5A), the f_basic _(red colour) in each second analyzed was kept consistent along the examination period. However, in terfenadine-treated embryo with bradycardia (Fig. 5B), the f_basic _was changed along time. In addition, some terfenadine-treated embryos had shown similar heart rate with control embryos (Fig. 5C), but the f_basic _was also shifted time by time. The rhythmicity index, as defined by the coefficient of variation in STFT power spectra, in control embryos was 0.03421 ± 0.0018 (Fig. 6). The rhythmicity index of terfenadine-treated embryos (including both embryos with bradycardia and with normal heart rate) was 0.07011 ± 0.0047 which was statistically significant different to the rhythmicity index in control embryos (*p *< 0.05).

## Discussion

This paper presents a simple and rapid method to analyze cardiac functions in small transparent animals, like zebrafish embryos, using video imaging technology.

### Sample preparation

Analysis of cardiac performance by digital motion analysis requires immobilization of animal samples because any movement could result in complication of subsequent data analysis. Therefore, immobilization is an important step for successful acquisition of video images. Immobilization of zebrafish embryo is commonly achieved by anesthetization, followed by mounting embryos in agarose of low concentration. Anesthetization of embryos was performed by incubation in 0.004% (w/v) triciane, equivalent to 40 mg/L, at which it was reported not to exhibit any adverse effect on heart rate and contractility [6]. The concentration used is lower than other studies have used [15,20], but is sufficient to suppress spontaneous body movement and successful acquisition of videos. On the other hand, higher concentrations of traciane, 50 mg/L [20] and 80 mg/L [15] also did not affect the cardiac performance in zebrafish embryos after anesthetization.

After anesthetization, embryos were mounted in low-melting point agarose at low concentration in order to hold them in fixed position for video acquisition. Liquid media is avoided because any fluid flow, even in slow speed, will cause errors in subsequent video analysis. Although oxygen equilibration is reduced in agarose, cardiac activity of zebrafish embryos is not affected as reported [20].

Positioning of embryos is an important step in the sample preparation for capturing video. In our preparation, the spontaneous positioning of zebrafish embryos in lateral position at the bottom of media facilitates the procedures for video imaging analysis of caudal blood flow. In addition, embryo samples prepared for the analysis using the current method can be oriented in random direction. In the original procedures of digital motion analysis, embryos were placed in horizontal position and thus movement of blood cells was along the axis parallel to the lines of CCD chip [20]. It was because the shifting vectors were derived from the sum of all dynamic pixels on horizontal lines. However, in the method presented in this paper, orientation does not affect the determination of blood cells velocity because the amount of dynamic pixels was used, which was relevant to the velocity of blood cells, although no absolute velocity of blood cells was derived from the dynamic pixels. Altogether, the procedures of sample preparation for the presented method are more effective and less time-consuming.

Milan and his co-works [8] had also used Fourier Transform to determine heart rate in 2-day old zebrafish embryos. The difference of their method with our present method is the region of interest. They analyzed the ventricle with Fourier Transform and used ventricular rate as the index of heart rate. However, as mentioned in Schwerte's paper [22], heart rate determined by Fourier Transform was inferred by movement from all tissues around the heart. Furthermore, in our experience, depending on view angle, yolk gland (still present at 2-day old embryos) could cover part of the heart, making analysis of the heart region with power spectrum analysis of periodic change in pixel intensity difficult. Therefore, we chose the caudal part as our region of interest for analysis. At this part, the only moving objects were blood cells clearly be visualized inside blood vessels.

### Determination of heart rate

Pulsatile movement of blood cell was observed in the caudal vasculature of zebrafish embryos and was revealed in the waveform pattern of dynamic pixels by our program. Heart rate determined by power spectral analysis and by visual counting was not statistically different, suggesting that the heart rate determined by either method was the same. In addition, the slope of regression line equals to 1 showed that the heart rates determined by power spectral analysis and visual counting are equivalent. The advantages of using power spectral analysis include rapid and full automation during the calculation procedures and researchers do not need to sit in front of the microscope to count heartbeat frequency.

To demonstrate the use of power spectral analysis in determination of heart rate from caudal vasculature, we applied the method in zebrafish embryos exposed to one of well known QT-prolonging drugs, terfenadine. Previous studies demonstrated that QT-prolonging drugs induced bradycardia in zebrafish embryos, which was similar to the physiological response in human [6,8]. Similarly, our method was also able to detect bradycardia in terfenadine-treated embryos. In addition to bradycardia, QT-prolonging drugs examined were able to induce arrhythmia [6].

### Determination of variability of heart beat interval

Rhythm of heartbeats described the regularity of heart beat. In mammal and avian, rhythmic change of heart rate is controlled by nervous system, even beginning from the embryonic stage to adult. For example, the observation of heart rate irregularities in chicken embryos suggested that embryonic heart was stimulated by nervous system starting from day 12/13 [26]. However, sudden deviation of heart rate from its baseline was, sometimes, lethal in human being. Sudden death was associated with heart rate arrhythmia [27-29]. For example, variability of QT interval was higher in dilated cardiomyopathy patients compared with control persons [30].

Terfenadine affected the ventricle more than the atrium in zebrafish embryo, resulting in the observation of 2:1 atrioventricular blockage. High degree of blockage was observed at higher concentration or extended incubation time, sometimes leading to fibrillation and irregular arrhythmia (i.e. the atrium and ventricle beating irregularly, not in 2:1 fashion) [6]. However, increase in the variability of heart beat interval in zebrafish embryos after terfenadine treatment was not reported in previous studies. We had demonstrated the increase of the variation of heartbeat detected in terfenadine-treated embryos. The poincare plot showed changes of time interval between two consecutive heart beats in both control and terfenadine-treated embryos beat by beat. The STFT analysis illustrated the changes of heart rate time by time which was similar to poincare plot, indicating the ability of our program to determine the variation of heart beat frequency. Moreover, the calculated coefficient of variation from power spectral analysis, which was determined as rhythmicity index, showed that our program was able to detect the changes of variation of heart rate in zebrafish embryos.

The variability of heart beat interval was not only determined from ECG but also from blood pressure. McKinley and co-workers demonstrated deriving reliable index of heartbeat variability from peripheral blood pressure waveform [31]. In their study, peaks and troughs of the waveform were marked, from which beat-to-beat intervals were determined. Their results showed that data derived from blood pressure waveform correspond to the data determined from ECG. Since determination of heart beat interval variability depended on the precise identification of peaks and troughs in waveform data, complex algorithm should be used for automatic detection and analysis from the data of blood pressure. In our experiments, manual adjustment was sometimes needed for more accurate determination of peak and trough prior to data analysis. However, detection of beat peak was not necessary in power spectral analysis. Instead, variability of beat-to-beat frequency was determined by the rhythmicity index in our program. Our results demonstrated that cardiac rhythm was able to determine from the direct measure of beat-to-beat interval variability and the power spectral analysis. The advantage of using power spectral analysis was that the results were determined from data covering a period of time of heartbeat and the analysis and determination were fully automated.

## Conclusion

In summary, we have developed a rapid, simple and non-invasive method to determine the heart rate and heartbeat regularity from the peripheral circulation of zebrafish embryo by digital motion analysis and power spectral analysis. We have demonstrated the ability of our method in determination of terfenadine-induced bradycardia and arrhythmia in 52-hpf embryos. With the advantages of rapid sample preparation procedures, automatic image analysis and data analysis, this method is applicable to study the molecular mechanism of variability of heart beat interval as well as to screen side-effect of cardiotoxicity of non-cardiovascular drugs.

## Methods

### Fish

Breeding colonies of zebrafish were obtained from local supplier and were kept in small aquaria according to conditions described in [32]. Briefly, they were kept at 28°C water and subjected to 14-hour light/10-hour dark cycle. Spawning occurred when the light was just turn on. Eggs were collected after 30 minutes of light turning on. Collected eggs were incubated at 28°C in embryo buffer (19.3 mM NaCl, 0.23 mM KCl, 0.13 mM MgSO_4_·7H_2_O, 0.2 mM Ca(NO_3_)_2_, 1.67 mM Hepes, pH 7.2). At 4-hour post fertilization (hpf), eggs were examined under dissecting stereo-microscope (SZX-12, Olympus, Tokyo, Japan). Dead or unfertilized eggs were removed. Only embryos which have developed normally and reached the blastula stage (30% epiboly) were selected for subsequent experiments.

### Treatment of cardiotoxic drug

At 4 hpf, 20 embryos were incubated in 6-ml embryo buffer containing 0.003% (w/v) 1-phenyl-2-thiourea (PTU) in a Petri dish. PTU at this concentration did not have any effect on zebrafish embryos except inhibiting the formation of pigment cells. Terfenadine, a cardiotoxic drug, was purchased from Sigma. Stock of terfenadine was prepared by dissolving in dimethyl sulfoxide (DMSO) with final concentration of 10 mM. At 50 hpf, 6-μl stock solution of terfenadine were added to 6-ml embryo buffer to final concentration of 10 μM. The final DMSO concentration in embryo buffer was 0.1% at which did not cause any adverse effect. Embryos were exposed to terfenadine for 2 hours prior to video recording.

### Imaging of caudal blood circulation

**Figure 7:**
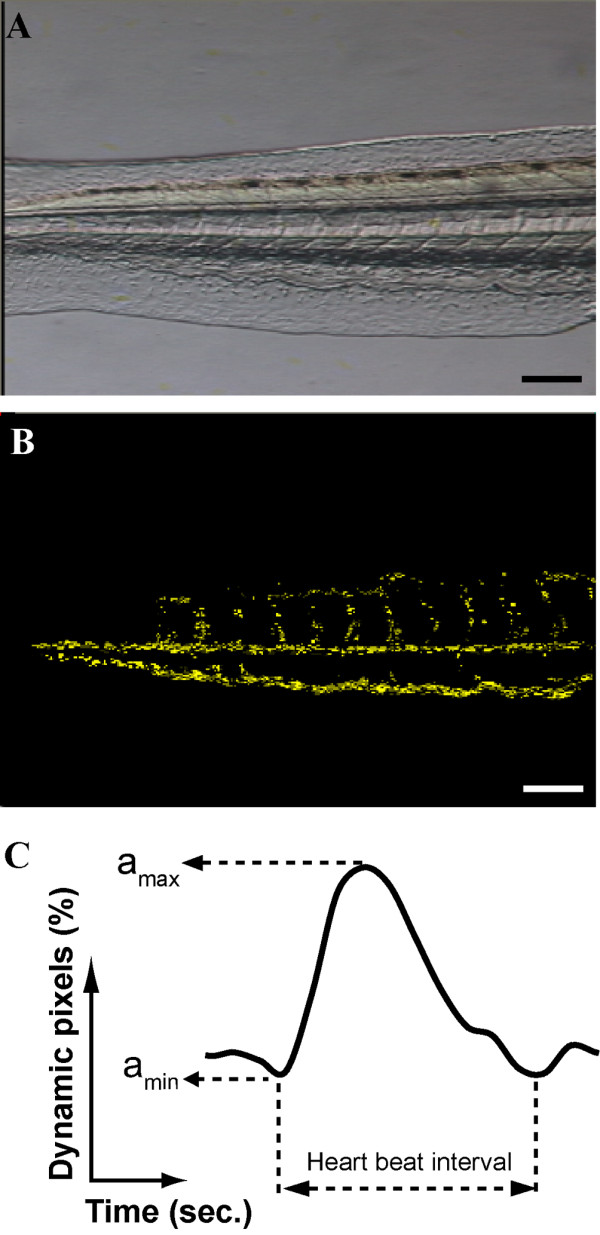
**Diagram showing the caudal region of embryo to be analyzed (A) and the result image of subtraction between 2 consecutive video frames (B) with yellow pixels representing the dynamic pixels**. Schematic diagram illustrated the parameters being analyzed in the signal of dynamic pixels (C). a_max_: maximum value of dynamic pixels; a_min_: minimum value of dyannmic pixels; heart beat interval: the time for a complete heart beat between 2 toughs; Scale Bar: 50 μm; Right to anterior (head).

To avoid the movement of zebrafish embryo during videoing, embryos were first anaesthetized by adding tricaine (MS-222) to embryo buffer at 52 hpf. The final concentration of tricaine in embryo buffer was 0.004% (w/v) which did not have any effect on heart beat in zebrafish embryos [6,21]. Embryos were then mounted laterally in 0.5% low-melting point agarose. To prevent dry out of agarose during videoing, a small aliquot of incubated embryo buffer containing tricaine was added on the top of agarose. Embryos were examined under stereo-microscope (Olympus) equipped with a 3-color CCD camera (DC330, Dage-MTI Inc., Michigan City, IN, USA), which in turn was connected to NTSC digital video recorder (Sony, Tokyo, Japan) via S-video cable. The video of blood flowing in caudal region (Fig. 7) was captured at 48× magnification for 1 min. Videos were stored in mini-DV tapes and were grabbed to personal computer by MovieMaker (Microsoft, USA) in AVI format.

### Digital motion analysis

A custom made program was developed in C# language (Visual Studio .NET, Microsoft) to implement the method of digital motion analysis developed by [20]. In brief, a software frame-grabber, based on the DirectShow developer tool kit in DirectX version 9 (Microsoft) was used to grab video frames one by one and convert RGB colored video frame to gray-scale image. Each image was subtracted by image of consecutive video frame pixel-by-pixel in gray-scale. The value of static pixels located in non-moving areas where pixel intensities between 2 images were not different was zero. Whatever there was difference in the intensity of particular pixel between 2 images, that pixel was counted as dynamic pixels and the intensity of that pixel in subtracted image was assessed as 255. Figure 7B illustrated a typical result after subtraction. To avoid identification of noise as dynamic pixels, threshold value of 10 was applied, i.e. the pixel was considered as dynamic pixel when the intensity difference between 2 images was greater than 10. Amount of dynamic pixels were expressed as the percentage to total number of pixels in the images, i.e. 720 × 480 pixels. Waveform of dynamic pixels was plotted for each subtracted video frames. Peaks (a_max _in Fig. 7C) and troughs (a_min _in Fig. 7C) of the waveform were identified automatically in the custom made program. The duration between the 2 a_min _was defined as the heart beat interval (Fig. 7C) which was equivalent to the time interval of a complete cycle of systole and diastole of ventricle.

### Power spectral analysis

Waveform of dynamic pixels was analyzed by Fourier transform (FFT) and power spectral analysis. Transformation and analysis were implement by C# language (Visual Studio .NET, Microsoft) using a custom made program. Function of Fourier transform was implemented by the derivation of Glassman n-point fast Fourier transform algorithm [33] which was original written in Fortran language. Power spectrum was calculated by power spectrum estimation algorithm described by [34]. To avoid data leakage during power spectrum estimation, Welch data windowing function was applied. Since video was recorded in frequency of 30 Hz, the maximum frequency, in principle, was the half of the data acquisition frequency, i.e., 15 Hz. Basic frequency component was calculated. Before transformation, bandpass filter (0.5 to 5 Hz) was applied to signal data.

#### Short-time Fourier Transform (STFT) analysis

Another analysis performed by the custom-made program is STFT analysis, which is a Fourier-related transform used to determine the sinusoidal frequency and phase content of local sections of a signal as it changes over time. Analysis was done on every 0.5 sec and the time interval for each analysis is 1 sec, i.e., overlapping of 0.5 sec in each analyzed data segment. Three parameters were calculated, including mean frequency, which is calculated by summation of frequency times power value of that frequency, standard deviation of frequency, which is the variation of the frequency along time, and the coefficient of variation, which is computed by dividing the standard deviation of frequency by the mean frequency. The coefficient of variation was reported as the rhythmicity index. The larger the value of the index, the more the variation of the heart rate is along time. Data extracted were exported to Excel (Microsoft) for statistical analysis.

### Poincare plot

Poincare plots, correlating observation *n *on the *x*-axis with observation *n+1 *on the *y*-axis, were used to demonstrat the beat-to-beat variation of the length of cardiac cycle. The heart beat interval was determined from the temporal plot of dynamic pixels. Each interval in the sequence was first *n *and then *n+1 *until each interval in the time series were plotted against its successor.

### Statistical analyses

Data were extracted and exported into ASCII text file from the custom made programs and pre-processed in Excel (Microsoft, USA). Statistical analyses were performed using GraphPad Prism 2 (GraphPad Software Inc., San Diego, CA, USA). Data were presented as mean ± SEM. Statistical comparison were made with a 2-sample *t*-test in 2-tail sided. Significance was accepted when *p *< 0.05. Correlation analysis was performed by Spearman method. Nonlinear regression analysis was performed with different models that compared between each other to find out the best fit one suggested by Prism.

## Abbreviations

PTU: 1-phenyl-2-thiourea; f_basic_: Basic frequency; DMSO: Dimethyl sulfoxide; FFT: Fourier transform; hpf: Hour post-fertilization; STFT: Short-time Fourier Transform.

## Authors' contributions

PKC performed drug treatment experiments, acquired videos, wrote the custom made software, analyzed videos, plotted the graphs and drafted the manuscript. CCL participated in the statistical analysis and revised the manuscript. SHC participated in the design and coordination of the study, and gave final approval of the version to be published. All authors read and approved the final manuscript.
